# Compositional analysis of the tonsil microbiota in relationship to *Streptococcus suis* disease in nursery pigs in Ontario

**DOI:** 10.1186/s42523-022-00162-3

**Published:** 2022-01-21

**Authors:** Maysa Niazy, Sarah Hill, Khurram Nadeem, Nicole Ricker, Abdolvahab Farzan

**Affiliations:** 1 Bioinformatics Program, College of Biological Science, University of Guelph, Guelph, ON Canada; 2 Department of Population Medicine, Ontario Veterinary College, University of Guelph, 50 Stone Rd E, Guelph, ON N1G 2W1 Canada; 3 Department of Mathematics and Statistics, College of Engineering and Physical Science, University of Guelph, 50 Stone Rd E, Guelph, ON N1G 2W1 Canada; 4 Department of Pathobiology, Ontario Veterinary College, University of Guelph, 50 Stone Rd E, Guelph, ON N1G 2W1 Canada

**Keywords:** *Streptococcus suis*, Tonsil microbiota, Nursery pigs, Modelling, Metacommunities

## Abstract

**Background:**

The tonsil of the soft palate in pigs is the colonization site of both commensal and pathogenic microbial agents. *Streptococcus suis* infections are a significant economic problem in the swine industry. The development of *S. suis* disease remains poorly understood. The purpose of this study was to identify whether the tonsillar microbiota profile in nursery pigs is altered with *S. suis* disease. Here, the dynamics of the tonsillar microbiota from 20 healthy pigs and 43 diseased pigs with *S. suis* clinical signs was characterized.

**Results:**

Based on the presence or absence of *S. suis* in the systemic sites, diseased pigs were classified into confirmed (n = 20) or probable (n = 23) group, respectively. Microbiota composition was assessed using the V3-V4 hypervariable region of the 16S rRNA, and results were analyzed to identify the diversity of the tonsillar microbiota. The taxonomic composition of the tonsil microbiota proved to be highly diverse between individuals, and the results showed statistically significant microbial community structure among the diagnosis groups. The confirmed group had the lowest observed species richness while the probable group had higher phylogenetics diversity level compared to the healthy group. Un-weighted Unifrac also demonstrated that the probable group had a higher beta diversity than both the healthy and the confirmed group. A Dirichlet-multinomial mixture (DMM) model-based clustering method partitioned the tonsil microbiota into two distinct community types that did not correspond with disease status. However, there was an association between *Streptococcus suis* serotype 2 and DMM community type 1 (*p* = 0.03). ANCOM-BC identified 24 *Streptococcus* amplicon sequence variants (ASVs) that were differentially abundant between the DMM community types.

**Conclusions:**

This study provides a comprehensive analysis of the structure and membership of the tonsil microbiota in nursery pigs and uncovers differences and similarities across varying *S. suis* disease status. While the overall abundance of *Streptococcus* was not different among the diagnosis groups, the unique profile of DMM community type 1 and the observed correlation with *S. suis* serotype 2 could provide insight into potential tonsillar microbiota involvement in *S. suis* disease.

**Supplementary Information:**

The online version contains supplementary material available at 10.1186/s42523-022-00162-3.

## Background

*Streptococcus suis* is a Gram-positive bacterium that is a common inhabitant of the nasal cavities and tonsil of swine [1]. *S. suis* is a zoonotic agent and considered an occupational disease that can affect people working in close contact with infected pigs or pork products [2–4]. Infections caused by *S. suis* result in considerable economical losses in the swine farming industry [5]. In fact, *S. suis* has been incorporated among the top ten pathogens in swine worldwide [6]. Another challenge is the main costs associated with *S. suis* disease treatment and control [5]. Commonly, antimicrobials including beta-lactams such as penicillin, ceftriaxone, and ceftiofur are used to control the disease [7]. *S. suis* is considered to be a reservoir of antimicrobial resistance genes due to the usage of antimicrobials [2, 8]. Also, there has been no universal vaccine available and autogenous vaccines are commonly used with contradictory outputs [2].

There are presently 29 serotypes of *S. suis* identified based on capsular polysaccharide antigens [9]. Most studies indicate that serotypes isolated from diseased pigs primarily belong to serotype 1–9, 1/2, and 14 with different distribution depending mainly on geographical regions [10]. So far, *S. suis*serotype 2 is most frequently recovered from the diseased pigs; however, the strains may vary genotypically and phenotypically between different continents [9]. While most pigs are asymptomatic carriers of the bacterium, approximately 5% of pigs develop clinical signs of *S. suis* infection with pigs at age 5–10 weeks being most susceptible to infection [11]. Usually, *S. suis* infections are seen in weaned piglets about 2–6 weeks after weaning, not often in suckling and growing animals, and rarely in adult pigs. Clinical manifestations of *S. suis* infection include fever, septicemia, which in turn can lead to meningitis, endocarditis and lameness due to arthritis [1]. Commonly, sow vaccination is used in the field to provide passive protection to the piglets. While it is less costly, it offers weak results due to the decline of maternal antibodies by 2–3 weeks post-weaning [1]. However, it is not fully understood why some pigs develop clinical signs of *S. suis* while others remain healthy carriers. One approach to understanding how *S. suis* can develop into infection is to look at the microbial environment of the tonsils. The tonsils of the soft palate play an important role in innate and acquired immunity and can provide an immediate defence mechanism against pathogens [12, 13]. Paradoxically, the tonsils are also an important niche for microbial colonization and a portal of entry for a variety of microbes including those that are pathogenic [13].

Previous studies have examined the tonsil microbiota of swine populations by describing the core microbiome and how the microbiome is developed through age. *Actinobacteria, Bacteroidetes, Firmicutes, Fusobacteria* and *Proteobacteria* have been reported as the 5 most predominant phyla in the tonsil microbiota in pigs [14, 15]. Several studies have examined the development of the tonsil microbiota in healthy pigs from newborn to 19 weeks of age and found that a change in the host environment, such as change in feed, temperature and medication can affect the microbial environment, potentially altering the presence of bacterial species abundance [15–17]. A study by Cortes et al. demonstrated that in-feed medication, the stress of changing diet, and movement from farrowing to the nursery room were associated with a shift in bacterial composition and affected the development of the microbiome [17].

Research investigating the interactions between the members of polymicrobial communities in the upper respiratory tract of swine is still in the early stages. However, culture-independent methods have recently revealed a complex consortium of microbes associated with respiratory diseases in swine [16] and identifying the combinations of organisms include bacteria, protozoans, viruses, and fungi [18]. Piglets are colonized by *S. suis* from vaginal secretions through parturition and during nursing [1]. This colonization may lead to an asymptomatic carriage or develop to an invasive disease, particularly in the subject of coinfections with porcine reproductive and respiratory syndrome virus (PRRSV), swine Influenza A Virus, or polymicrobial infections [19].

The presence of multiple bacterial and viral pathogens may cause porcine respiratory disease complex (PRDC) [20]. While *Actinobacillus minor* and *Actinobacillus porcintonsillarum* are benign commensals, *Actinobacillus pleuropneumoniae, Mycoplasma hyorhinitis,* and *Mycoplasma hyopneumoniae* are known as swine pathogens [21]. Coinfections and combinations of causative infectious agents are frequently occurred in respiratory diseases in pigs [22]. A previous study demonstrated that highly virulent influenza A virus (IAV) infection promotes *S. suis* adherence, colonization and invasion [23]. Another study has shown that pre-infected bone marrow derived dendritic cells with PRRS virus can amplify expression of pro-inflammatory genes caused by *S. suis* infection [24]. Pathogen-pathogen-host interactions associated with *S. suis* infection are also reported. A recent study showed that co-infection with *B. bronchiseptica* may increase the colonization and the cytotoxic effects of *S. suis* [25]*.* In fact, the interactions occurring in the microbial communities could facilitate the pathogen survival in host systems [16, 19, 22–25]. In this study, pigs were not tested for presence of other respiratory bacterial and viral pathogens, however, the sick pigs were identified based on typical clinical signs of *S. suis* infection and confirmed by isolation of *S. suis* from systemic sites. The objectives of this study were (i) to characterize the tonsillar microbial communities of healthy pigs and those developing clinical symptoms of *S. suis* infection, (ii) to model the association between the tonsil microbiota composition and clinical *S. suis* disease.

## Results

Two samples were removed due to low reads resulting in samples from 63 pigs classified into three diagnostic groups including 20 confirmed, 23 probable, and 20 healthy pigs. A summary of the number of confirmed, probable, and healthy pigs before and after quality control is included in the supplemental material (Additional file 3: Table S1). A total of 5,733,407 raw reads were processed, with an average of 91,006 reads per sample (median 92,001) and a total of 12,921 amplicon sequence variants (ASVs).

### Diversity analysis

**Fig. 1:**
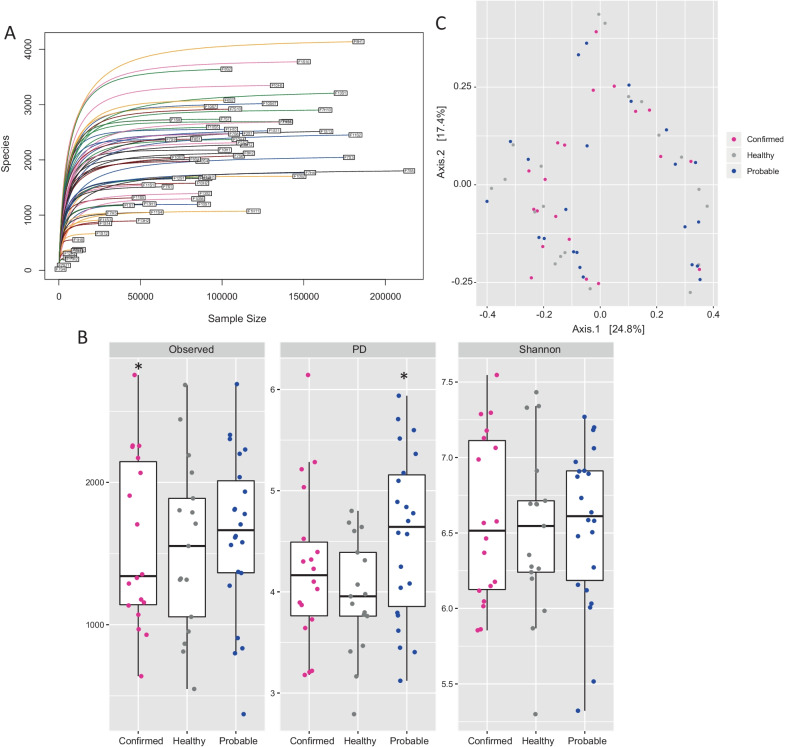
**A** Rarefaction curves that estimate microbial diversity of three diagnosis groups. The lines represent the different sampling depth across the samples. **B** Phylogenetics (PD) and non phylogenetics (Observed ASVs and Shannon) alpha Diversity metrics among the 57 rarefied samples (18 confirmed, 17 healthy, and 22 probable cases) on 9 swine farms. The probable group had the highest level of phylogenetics diversity while the confirmed group had the lowest observed ASVs. Asterisks indicate significant differences between diagnosis groups *p < 0.05. **C** Non-metric multidimensional scaling (NMDS) plot of Bray–Curtis dissimilarity distance index for 57 rarefied samples (18 confirmed, 17 healthy, and 22 probable pigs) on 9 swine farms

The rarefication curves showed sufficient sequence depth for all samples and that the within samples diversity was fully captured (Fig. 1a). Using the Kruskal‐Wallis test, there was no statistically significant difference among pigs in three groups detected based on the Shannon index (*p* = 0.2) (Additional file 3: Table S2). However, as shown by species observed index the confirmed group had the lowest richness species compared to the healthy (*p* = 0.051) and the probable (*p* = 0.038) groups (Fig. 1b). Based on Faith Phylogenetic diversity that assessed using the Tukey Honest Significant Difference method, the probable pigs had a higher phylogenetic diversity level compared to healthy pigs (*p* = 0.045) and showed a trend towards greater diversity than the confirmed group (*p* = 0.07) (Fig. 1b). Based on PERMANOVA, beta-diversity was not significantly different between the three groups based on Bray–Curtis distances (*p* = 0.22) (Fig. 1c); however, un-weighted Unifrac demonstrated that the probable group had the highest phylogenetics diversity compared to healthy (*p* = 0.014) and confirmed group (*p* = 0.019), consistent with the increased alpha diversity observed for this group.

### Taxonomic composition and core tonsillar microbiota

**Fig. 2:**
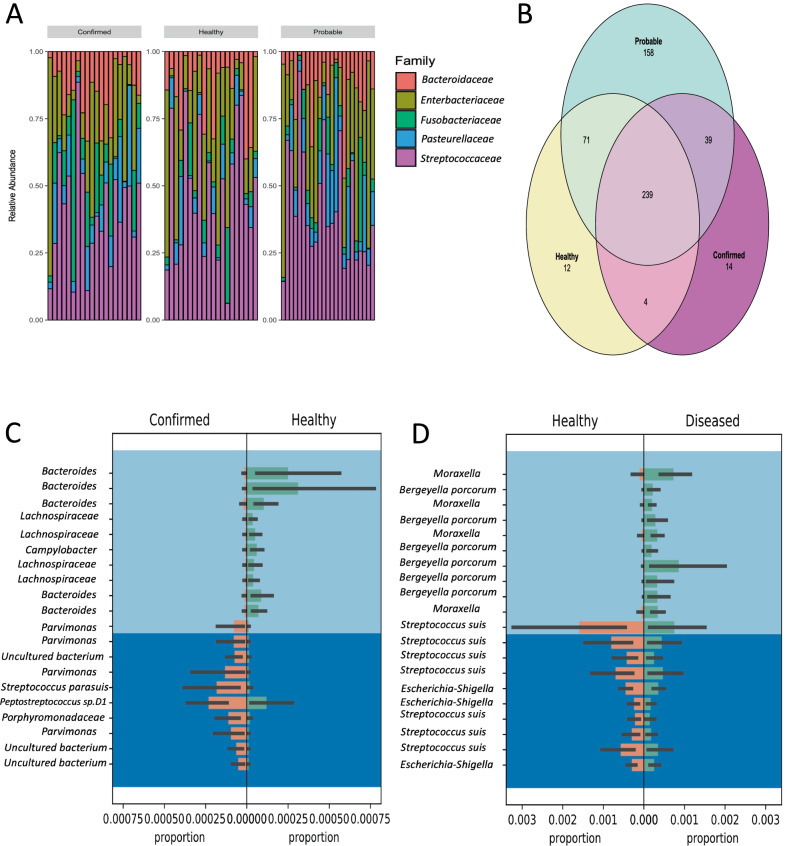
**A** Relative abundance of bacterial families detected in the diagnosis groups (20 confirmed, 20 healthy, and 23 probable cases) on 9 swine farms. *Streptococcaceae* had the higher relative abundance. **B** Three-way Venn diagram of the microbial ASV composition in the tonsillar microbiota of the confirmed, probable, and healthy groups. Percentages represent either unique or shared ASVs across the diagnosis groups. **C** Balance Trees generated using the Gneiss tool identified high abundance of *Peptostreptococcus sp. D1, Streptococcus parasuis,* and *Parvimonas* in the confirmed group while *Bacteroides, Lachnospiraceae* and *Campylobacter* were found to be higher in the healthy group. **D **The proportion plot of the abundance between *Streptococcus suis, Moraxella,* and *Bergeyella porcorum* based on Gneiss proportions for individual ASVs found to differ between the healthy and the combined diseased (confirmed and probable) cases. The top light blue represents numerator taxa, and the bottom dark blue represents denominator taxa

There were six phyla with over 1% relative abundance in all groups, which accounted for 98.7% of the total sequences including *Firmicutes, Proteobacteria, Bacteroidetes, Fusobacteria, Tenericutes*, and *Actinobacteria*. Also, there were four phyla with less than 1% relative abundance including *Chlamydiae, Epsilonbacteraeota, Spirochaetes,* and *Patescibacteria* (Additional file 1: Fig. S1a). At the family level, *Streptococcaceae, Enterobacteriaceae*, *Pasteurellaceae, Bacteroideaceae,* and *Fusobacteriaceae* were found to be the top five families which accounted for 75% of the total sequences (Fig. 2a)*.* The two most predominant genera were *Streptococcus* and *Escherichia-Shigella,* which accounted of 52% of the total frequency of the sequences (Additional file 1: Fig. S1b)*.* While the taxonomic classification analysis was able to characterize 93% of the sequences to the family taxonomic level, only 84% of the sequences were assigned to the genus level.

Our results showed that *Streptococcus* genus constitutes the largest proportion (32.7%) of the total reads, and within *Streptococcus* ASVs, the species *Streptococcus suis* was the most abundant and accounted for 25.7% of the total sequences with the highest frequency found in the healthy group (29.9%). Consistent with this finding, permutational multivariate analysis of variance (PERMANOVA) and the homogeneity analysis indicated that *Streptococcus* ASVs abundance were not different between the probable and the healthy groups (*p* = 0.42) but tended to be higher in the healthy group compared to the confirmed group (*p* = 0.09).

Among the 12,921 ASVs, 537 were core taxa and present in 50% of all the samples. Out of these bacterial taxa, 239 ASVs were shared by the confirmed, healthy, and probable groups, accounting for ~ 45% of the core tonsil microbiome (Fig. 2b). Over half of the shared ASVs (52%) across the three diagnosis groups were belonged to four families: *Streptococcaceae, Enterobacteriaceae, Bacteroidaceae* and *Pasteurellaceae*, with *Streptococcus, Escherichia-Shigella, Bacteroides* and *Actinobacillus* being the most shared genera. The healthy group shared 95% of the core ASVs with the probable group while shared 74.5% with the confirmed group.

Using Pairwise PERMANOVA analysis (permutations = 999), there was no difference among pigs in three diagnosis groups at the phylum and family levels (*p* = 0.48, *p* = 0.15 respectively). At the genus level, there was no difference between the healthy and the probable groups (*p* = 0.17) and between the confirmed and the probable group (*p* = 0.28). However, the taxonomic composition of the confirmed group was different compared to the healthy groups (*p* = 0.016). PERMANOVA analysis identified 15 of the 79 genera as the most importance taxa since they had higher correlation coefficients and high contribution to the differences in the tonsillar microbiota (Additional file 1: Fig. S1.c). Using Gneiss balance trees, *Streptococcus parasuis, Peptostreptococcus sp. D1, Porphyromonas,* and *Parvimonas* were found more abundant in the confirmed group while *Bacteroides*, *Lachnospiraceae*, and *Campylobacter* were found to be more abundant in the healthy group (Fig. 2c). In addition, there was a balance, referring to the log ratios between the species, between the abundance of *Streptococcus suis, Escherichia*-*Shigella, Bergeyella* and *Moraxella*. As shown in the proportion plot (Fig. 2d), *Moraxella* and *Bergeyella porcorum* were found to be higher in the diseased group (confirmed and probable cases) while *Streptococcus suis* and *Escherichia*-*Shigella* were found to be higher in the healthy group.

### Clustering and microbial community structure

**Fig. 3:**
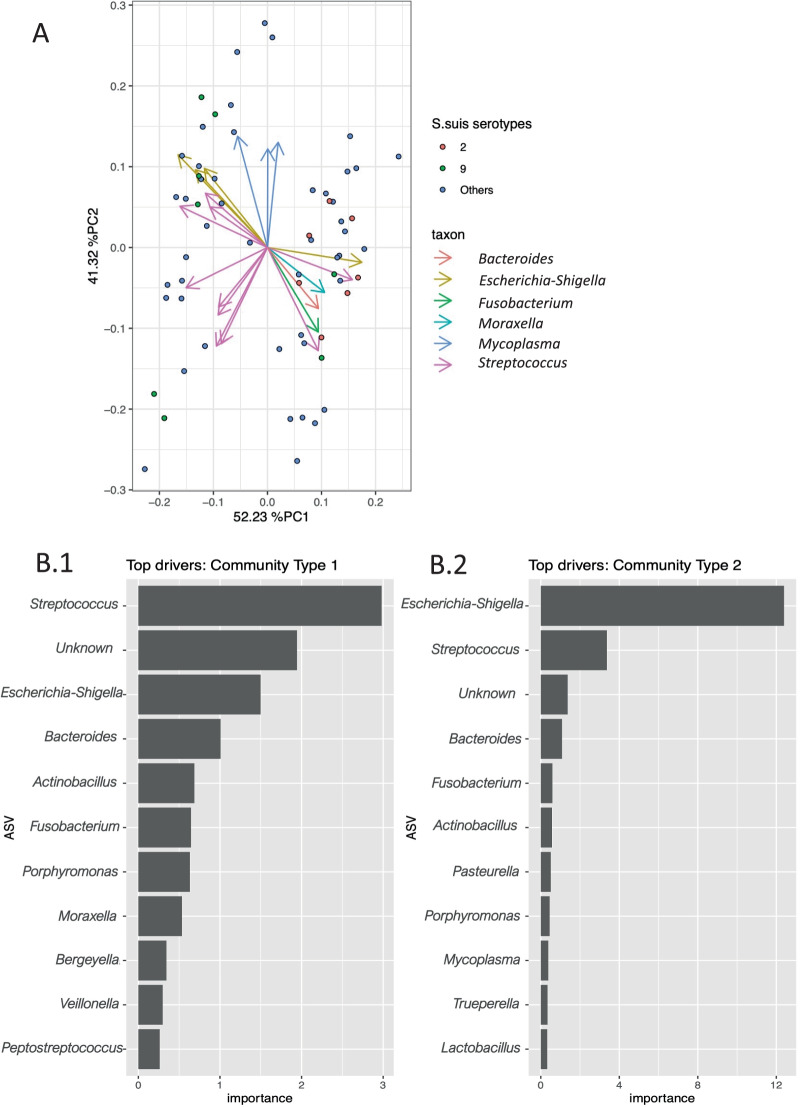
**A** DEICODE biplot using Aitchison distance. The principal components represent ~ 93% of variance. Principal component 1 (PC1) is negatively correlated with the abundance of *Streptococcus* but positively with *Moraxella, Fusobacterium,* and *Escherichia-Shigella* while principal component 2 (PC2) is positively correlated with *Escherichia-Shigella, Mycoplasma*, and negatively with *Streptococcus*. The arrow directions point to the increased log ratio between the features. Others refers to other observed *S. suis* serotypes (3, 4, 5, 7, 8, 10, 11, 12, 15, 16, 28, 29, 30 and 31). **B** The DMM model community types. **B.1** the variable importance and the leading taxa of community type one that dominated with *Streptococcus* along with *Moraxella, Bergeyella,* and *Veilonella*. **B.2** the variable importance and the leading taxa of community type two that dominated with *Escherichia-Shigella* along with *Pasteurella, Trueperella,* and *Mycoplasma.* The values of importance are scaled to relative abundance and transformed to square root

The non-metric distance scaling (NMDS) analysis using both the un-weighted Unifrac matrix, and Bray–Curtis dissimilarity revealed two clusters based on the bacterial profile that were not associated with the diagnostic groups (Fig. 1c). Therefore, a log-ratio principal-component analysis (PCA) based method was used to explore those clusters and to investigate underlying structural differences in the microbial community structure regardless of the pigs’ disease status. The PCA using the Aitcheson matrix explained more than 93% of the variance in the data (Fig. 3a), and the principal component 1 was negatively correlated with the abundance of *Streptococcus* but positively with *Moraxella, Fusobacterium,* and *Escherichia*-*Shigella*. Using serotype information available for these samples, we also investigated the correlation between distribution of *S. suis* serotypes and community structure. While *S. suis* serotype 2 was correlated only with principal component 1, serotype 9 was fairly distributed across the two principal components (Fig. 3a). Principal component 2 was positively correlated with *Escherichia-Shigella* and *Mycoplasma* but negatively with *Streptococcus.* The DMM community typing analysis further supported the patterns seen by NMDS and PCA. The DMM model partitioned the data samples into two distinct community types (clusters based on microbial community profiling) neither of which was correlated with diagnosis groups.

Using chi-square test, the farm and age of pigs were not significantly associated with the health status of the pigs (*p* = 0.1 and *p* = 0.6, respectively) (Additional file 2: Fig S2.a/S2.b). Community types obtained from the DMM model were also not associated with farm, age or sex variables. While using chi-square test, *S. suis* serotype 2 was significantly associated with community type 1 (*p* = 0.03) (Table 1)*. Streptococcus*, *Fusobacterium,* and *Actinobacillus* were found in both community types and associated with a unique relative abundance profile in each community (Additional file 3: Table S3). Community type 1 was dominated by *Streptococcus* and was more influenced by *Moraxella*, *Bergeyella,* and *Veilonella* (Fig. 3b.1), while community type 2 was dominated by *Escherichia-Shigella* and *Bacteroides* and associated with *Pasteurella*, *Trueperella,* and *Mycoplasma* (Fig. 3b.2). Further, the analysis of composition of microbiomes with bias correction method (ANCOM-BC) identified 193 ASVs that were differently abundant between the two DMM community types (including 24 *Streptococcus* ASVs, *Actinobacillus indolicus, Glaesserella parasuis, Porphyromonas, Trueperella pyogenes,* and *Escherichia*-*Shigella* and *Mycoplasma*) (Additional file 3: Table S4).

**Table 1:** The distribution of *S. suis* serotypes recovered from tonsil of pigs across the two identified Dirichlet-multinomial mixture (DMM) model community types

*S. suis* serotypes	Number of isolates	
	Community type 1 (dominated by *Streptococcus, Moraxella, Bergeyella,* and *Veilonella*)	Community type 2 (dominated by *Escherichia-Shigella, Pasteurella, Mycoplasma,* and *Trueperella*)
2	8	0
3	1	1
4	1	0
5	1	0
7	1	0
8	1	1
9	2	7
10	1	0
11	4	1
12	1	0
15	1	3
16	3	3
18	0	0
21	0	1
28	5	1
29	2	2
30	1	0
31	2	1
Untypable	6	2
Total	41	23

## Discussion

The purpose of this study was to examine whether development of *S. suis* disease in nursery pigs is associated with structural changes in the tonsil microbiota. In particular, the tonsil microbiota of pigs with clinical signs of *S. suis* infection with presence (confirmed cases) and absence (probable cases) of *S. suis* in systemic sites and healthy control pigs was determined and the relationship between the microbiota composition and *S. suis* disease was analyzed.

The tonsils of the soft palate are secondary lymphoid organs which are involved in both innate and adaptive immunity. They produce mucin to prevent bacterial adherence, have physical barriers consisting of the epithelium and lumen of the crypts, produce antimicrobial peptides (AMPs), and trap microorganisms using phagocytes [12, 13]. Despite having a dynamic defense system, microbes are still able to colonize the tonsils. Bacterial pathogens including *Actinobacillus pleuropneumoniae*, *Glaesserella (Haemophilus) parasuis, Pasteurella multocida*, *Mycoplasma hypopneumoniae, *and* Mycoplasma hyorhinitis* are often present in swine tonsils [26]. *Pasteurellaceae*, *Streptococcaceae,* and *Moraxellaceae*—among the top families identified in the current study—have previously been reported as the most frequent and core families in the tonsil microbiome [14, 15]. *Pasteurellaceae* and *Streptococcaceae* are most likely inherited from the vaginal tract during birth while *Moraxellaceae* were most likely inherited from teat skin [15].

Tonsil microbial communities were found to be highly diverse, with abundant ASVs being shared among diagnosis groups. Based on PERMANOVA analysis, the confirmed group had lower species richness and was significantly different at the genera level from the healthy group. In addition, several taxa were differently abundant between the confirmed and the healthy group. *Peptostreptococcus sp. D1, Streptococcus parasuis*, *Campylobacter, Porphyromonas,* and *Parvimonas* were all higher in the confirmed group. However, the healthy pigs were found to have more *Bacteroides* and *Lachnospiraceae* which are considered beneficial commensals and have been associated with healthy piglets in previous research [27]. The colonization of tonsils with these beneficial commensal bacteria may play a role in preventing *S. suis* disease and should be investigated further. In addition, the lowest number of shared ASVs was found between the confirmed and the healthy group, suggesting that there was a considerable discrepancy in the community structure between these two groups.

The lack of significant difference in the abundance of *S. suis* ASVs between the confirmed and the healthy cases in this study could partly be due to the variability introduced by sampling from multiple farms, as there might be a higher bacterial similarity between pigs within a single farm than between pigs from different farms. The nasal microbiota has been shown to be driven by sow contact [28] and then undergo a large transformation within the first 7 weeks of life and is not stabilized until 2 to 3 weeks post weaning (age of 5 to 6 weeks) [29]. Different management practices such as ventilation, pig density, farm health status, pig flow, feeding program, in-feed medication as well as pig genetics and age of pigs might have had an impact on the tonsil bacterial community in samples collected from multiple farms in this study. Inclusion age and farms as variables didn’t explain the variation of the tonsil community composition (Additional file 2: Fig. S2.a/S2.b). As antibiotic use was not provided by three farms -accounting for 40% of the samples- it was not possible to include in water/feed medication to data analysis (Additional file 2: Fig. S2.c/S2.d). Post-weaning pigs have been found to have an increase in *Streptoccaceae* but a decrease in *Moraxellaceae* in the tonsil microbiome, mostly due to carbadox usage [17]. In our study, there was a “balance” identified between the abundance of *Streptococcus* and *Moraxella* which showed an inverse relationship between these two taxa in sick and healthy cases, suggesting that shifts in the relative abundance of these two taxa may be informative to health status. However, use of antibiotics could not be factored into this analysis due to unreported data. More importantly, gneiss is not designed to infer differences in abundance for each taxon, instead, it can show whether the absolute abundances of species in the numerator have decreased or increased comparing to those in the denominator on average [30].

Complementary to previous research, the results of this study demonstrate that the tonsil microbiota community is highly diverse and rich in bacterial species [14]. The tonsil microbiota in probable cases, defined as animals showing clinical signs of *S. suis* infection but without recovery of *S. suis* from systemic sites, had higher phylogenetics diversity compared to that found in healthy and confirmed groups based on the un-weighted Unifrac analysis. A phylogeny-based metric, un-weighted Unifrac is described as a qualitative measure of beta diversity [31] as it not targeting the differences in taxa abundances but is instead designed to detect differences in the presence or absence of taxa in different communities. Further evidence of the striking diversity of the probable group is that it had the largest number of unique ASVs. However, it is not clear why the probable group shared less ASVs with the confirmed group than those with the healthy group. Since antibiotic use was not recorded in the study it is possible that the higher diversity and lack of *S. suis* in systemic sites could be the result of various treatment regimens on different farms initiated prior to sampling. While PERMANOVA and core ASVs analysis found that the diagnosis groups had different observed ASVs and structure, pairwise comparisons did not detect differences between the three diagnosis groups, suggesting a relative consistency between tonsillar microbiota in different disease groups. It is worth mentioning that the farm samples were collected at different times, and the sampling time might be a factor driving microbial community differences, as more or less taxa might have been present during cleaning and/or feeding.

Given that the occurrence of *S. suis* disease is multi-factorial [32], our analysis, in particular the characterization of the probable group, support a likely relationship between tonsillar community structure and *S. suis* colonization or presence of other respiratory pathogens in the tonsil; therefore, investigating the presence of *S. suis* co-infection with various respiratory viruses such as influenza A virus (IAV) and porcine reproductive and respiratory syndrome (PRRS) virus is vitally important.

Unlike operational taxonomic units ((OTUs) identified by clustering at 97% similarity), the analysis of ASVs (identified by 100% similarity) in this study, elucidated the importance of strain diversity for understanding bacterial communities. It has also been illustrated that the pig tonsil can be colonized simultaneously with different S*. suis* serotypes [33]. As an initial step, the analysis was ran using OTUs clustering method which lacked the ability to reveal the differences between the groups of study (data not shown) [34]. In fact, the similarity of 97% to identify the OTUs might have the potential to lose the resolution and may have accounted for the observed differences between methods [35]. The diversity of *S. suis* ASVs identified in this study suggests that species or strain level differences are likely important to understanding of the factors contributing to their differential abundance and to disease development.

In this study the DMM clustering model, which is based on the community profile of the samples, resulted in two distinctive metacommunities that did not correlate with disease status. In each of those two communities *S. suis* was associated with different taxa. Further, *S. suis* serotype 2 was strongly correlated with community type 1 that was also associated with higher abundance of *Moraxella, Bergeyella,* and *Veillonela.* This community structure might suggest potential interspecies interactions and/or a co-occurrence relationship between the community members and *S. suis* serotype 2. This is further supported by the finding that *Moraxella* and *Bergeyella* had a stronger association with diseased animals compared to healthy. Associated bacteria may facilitate colonization by certain strains of *S. suis* or pave the way for *S. suis* to shift from a commensal bacterium to an invasive pathogen and develop *S. suis* disease. It is likely that these taxa are interacting through one of the numerous interspecies relationships and that the taxa of community type 1 may be important for the colonization of serotype 2 strains. For instance, it is well documented that there is a symbiotic relationship of oral biofilm formation between *Veillonella* and *Streptococcus* species in human oral cavities. *Streptococcus* species produce lactate which can be utilized by *Veillonella,* resulting in biofilm production [36], which is important for microbe-microbe interactions in their host environment [37]. It is possible that the increased abundance of certain taxa in community type 1 could enhanced *S. suis* serotype 2 colonization and confer a higher risk to develop *S. suis* disease. Since serotype 2 is most frequently recovered from the diseased pigs [9], understanding the host and environmental factors that promote community type 1 is critical to developing strategies to prevent the colonization of serotype 2 strains.

On the other hand, abundance of *Escherichia*-*Shigella* and association with *Pasteurella* and *Mycoplasma* were found to be taxonomic characteristics of community type 2. *Pasteurella* and *Mycoplasma* both are an opportunistic invaders commonly found in the swine tonsil [38]. The combination of *Pasteurella* and *Mycoplasma* has been frequently reported in several studies and associated with a decrease in the phagocytic abilities of the pig’s macrophages when the two pathogens are present, indicating a potential interaction between these pathogens [38, 39]. *Mycoplasma* could also suppress the phagocytic responses in alveolar macrophages when pigs were exposed to *Actinobacillus* [40]. This suppression may explain a susceptibility of the pig with *Mycoplasma* to a secondary bacterial infection [40]. The presence of both *Pasteurella* and *Mycoplasma* in community type 2 may indicate cooperation between these community members.

As shown in a study of the human microbiome, metagenomic analysis of the DMM metacommunities might also help to illustrate differences in the microbiota of each community type at the functional gene level [41]**.** Future studies are needed to explore functional differences within tonsil communities and to further examine the co-infection dynamics of *S. suis* with various bacterial taxa and respiratory viruses. Also, incorporating additional metadata such as antibiotic exposure, farm management, and pigs’ genetics could uncover more correlations between disease status and the tonsil microbial community.

## Conclusion

In this study, the composition of the tonsillar microbiota of healthy and diseased pigs was reported.

Significant differences in bacterial community composition, observed taxa, as well as phylogenetic diversity were found across the three diagnosis groups. Several bacterial members were shown to be significantly altered in animals depending on the health status of the animal including *Streptococcus parasuis, Peptostreptococcus sp. D1,* and *Parvimonas*. The tonsil microbiota of both healthy and diseased pigs was dominated by *Streptococcus* ASVs. The health status of the individual animal did not provide an explanation of the two community types obtained from the DMM model; however, the unique profile of community type 1 and the observed correlation with *S. suis* serotype 2 could reveal new insights on the tonsil microbiota interactions and their influence on *S. suis* infection*.* This model identified key taxa such as *Mycoplasma, Pasteurella multocida, Moraxella, Veillonella, and Bergeyella* that should be the focus of future research for their role in establishing tonsillar communities.

## Methods

All animal research performed in this study was approved by the Animal Care Committee at the University of Guelph.

### Farm and pig selection

Nine farms in Southwestern Ontario, Canada with on-going *S. suis* outbreaks were identified and visited over a period of 16–18 months. On each farm, pigs with clinical signs of *S. suis* infection including ataxia, tremors, opisthotonos, paralysis, dyspnea, convulsions, nystagmus, lameness, erythema, and paddling were selected (cases). On the same farm, healthy pigs were selected from a group of low value pigs that were unthrifty or which had problems such as hernias or rectal prolapse but did not exhibit clinical signs of *S. suis* (controls)*.* Depending on the frequency of on-going *S. suis* cases at each farm, some farms were visited multiple times. In total, 65 (45 sick and 20 healthy) pigs at age of 3–9 weeks were selected on 9 farms. In this study, pigs with clinical signs of *S. suis* disease were matched with the healthy controls from the same farm which likely represent the tonsil microbiota prior to disease development. The controls (11 F, 8 M), confirmed (6 F, 13 M) and probable (10 F, 12 M) were at age of 3–7 weeks, 3 to 9 weeks, and 3 to 7 weeks, respectively. A summary of the number of pigs per farm and the number of visits can be found in (Additional file 3: Table SI).

### Sample collection

The samples collected from sick pigs included meningeal swabs, blood samples, nasal and rectal swabs and tissue samples from heart, lung, liver, ileum, and tonsil of the soft palate. The meninges were swabbed (BBL culture swab) carefully to avoid potential contamination. Nasal and rectal swabs (BD E-swab) as well as tissue samples from tonsils of the soft palate and ileum samples were collected from healthy pigs. All samples were maintained at 4 °C until brought back to the laboratory. Tissues and swabs were stored at -20 °C until further processing.

### *S. suis* isolation, identification, and serotyping

All samples were cultured for *S. suis* as described previously [42]. Briefly, tissues, swabs and blood were streaked on phenylethyl alcohol blood agar plates and incubated for 48 h at 35 °C in an atmosphere of 5% CO_2_. The suspected isolates were sub-cultured on Columbia blood agar plates (OXOID, Ottawa, Canada) and incubated for 48 h at 35 °C. Bacterial DNA was extracted from *S. suis* suspected colonies using the InstaGene Matrix kit (Bio-Rad) following the manufacturer’s instruction. The presence of glutamate dehydrogenase (*gdh)* and recombination/repair protein (*recN)* gene was tested by PCR. The isolates were confirmed as *S. suis* if both *gdh* and *recN* genes were identified, [33]. The *S. suis* isolates were subjected to whole genome sequencing and serotyped by in-silico pipeline SsuisSerotyping_pipeline [43].

A pig with clinical signs of *S. suis* disease was considered as “confirmed” case if *S. suis* was isolated from at least one systemic site (blood, spleen, lymph node, and meninges), or as “probable” case if it was diagnosed with *S. suis* infection in the farm and *S. suis* could not be recovered from any systemic site (Additional file 3: Table SI).

### DNA extraction from tonsil tissues

The tonsil tissue was subjected to DNA extraction using the Qiagen DNeasy Blood and Tissue DNA extraction kit following the Gram-positive protocol. An initial check of the quality of the DNA was performed using a NanoDrop spectrophotometer (Thermo Fisher Scientific). The quantity of the DNA was checked using a Qubit fluorimeter (Thermo Fisher Scientific). However, the NanoDrop can over-estimate DNA concentration and the Qubit doesn’t automatically provide information about impurities in DNA samples; factors which can negatively affect sequencing results [44]. Therefore, both methods were used for more accurate quantification analysis of the DNA samples.

### 16rRNA sequencing

16S rRNA gene **(**V3-V4 region) libraries were prepared at the Advanced Analysis Center at the University of Guelph, following Illumina’s 16S metagenomic sequencing library preparation protocol. Sixty-five libraries were then sequenced using Illumina MiSeq technology (Illumina Inc) with Illumina MiSeq version 3 (paired-ends with 300 bp reads).

### Bioinformatics and statistical modeling

Amplicon sequences were processed through the Quantitative Insights Into Microbial Ecology (QIIME2 version 2020.8) pipeline [45]. Paired end reads were assembled and assigned to amplicon sequence variants (ASVs) using the Divisive Amplicon Denoising Algorithm 2 (DADA2) [46] implemented through QIIME2 with quality filtering (Phred ≥ 20) and chimera removal (default setting). Multiple sequence.

alignment (MSA) was performed with MAFFT and then filtered to mask highly variable reads [47]. MAFFT is a MSA tool version 7 which is able to handle the direction in nucleotide alignment and combine unaligned reads into an existing alignment [47]. FastTree 2 was employed to generate and root a phylogenetic tree [48]. Taxonomic classification was performed by training a Naive Bayes classifier on the Silva rRNA Database Project Classifier V3/V4 region [49]. Filtering was performed to remove reads assigned to non-bacterial domain. In addition, singleton and doubleton reads were removed from the downstream analysis. The distribution of the taxa across the diagnosis groups was investigated using microbiome package and the diagrams were constructed using Venny 2.1.0 [50].

To analyze alpha and beta diversity metrics, the sequence reads were rarefied at a depth of 11,041 using repeated rarefaction [51] resulting in 12,747 ASVs. Alpha diversity was measured using the species observed and Shannon indices for each sample, and the parameters were compared in the three diagnosis groups (confirmed, probable, healthy) using the Kruskal‐Wallis test and Tukey Honest Significant Difference method. Phylogenetic relationships among identified ASVs were calculated using Faith’s Phylogenetic Diversity (Faith 1992) using the rooted phylogenetic tree obtained from FastTree 2 tool [52]. Beta diversity was evaluated using Bray–Curtis and un-weighted UniFrac distances, and a non-metric distance scaling (NMDS) plot was used to represent the relationships between samples. PERMANOVA analysis was performed to assess beta diversity and to identify the factors shaping the dynamics of the tonsil microbiome using the adonis function in the R “vegan” package (v. 3) [53]. Homogeneity of multivariate dispersions analysis was performed using the betadisper function from vegan package. For the previous analysis, P-value is considered as significant if it is equal to 0.05 or lower. A log-ratio based principal-component analysis (PCA) designed for compositional data was performed using DEICODE method that does not require any rarefying step [54]. DEICODE was used to explore the association of the microbial communities’ composition with the explanatory variables including diagnosis groups (confirmed, probable, and healthy) and different *S. suis* serotypes. The confirmed and probable cases were combined into one group (designated ‘diseased’) in order to identify features that were differently abundant and related to clinical signs of *S. suis* infection. Differential abundance analysis using balance trees was performed between the diseased and the healthy pigs using Gneiss implemented through QIIME2 with the default hierarchical cluster parameters. Correction for multiple comparisons and coefficients with false detection rate with corrected *p*-values less that 0.05 are discarded [30]. A Dirichlet multinomial mixture (DMM) model, a probabilistic based method for community clustering of microbial population profiling, was used to identify subgroups or metacommunities with a similar composition [55]. The advantages of using DMM model is that DMM model can determine the optimal number of community types that fit the data best and identifies the contribution of each taxonomic group to each component. Chi-Square test of Independence was used to identify potential association between the community types and the exploratory variables. Differential abundance analysis was conducted at the aggregated Genus level to identify possible differences between the metacommunities using analysis of composition of microbiomes with bias correction method (ANCOM-BC) that applies the Wilcoxon rank test to find the significance variables [56].

## Supplementary Information

**Additional file 1**. **Figure S1:** Relative abundance of tonsillar microbiota of the diagnosis groups (20 confirmed, 20 healthy, and 23 probable cases) on 9 swine farms. **A**: Boxplots of the top 5 phyla show that Firmicutes had the highest relative abundance across the samples. Firmicutes outliers included two confirmed cases on two different farms and one healthy pig on another farm. **B**: Boxplots of the relative abundance of top 5 genera show that *Streptococcus* had the highest relative abundance across the three diagnosis groups. *Streptococcus* outliers include two confirmed cases from two different farms and one healthy pig on another farm. **C**: Model coefficients of the PERMANOVA analysis. Only the top 15 taxa that contribute most to the tonsillar community differences are shown. The genus *Streptococcus* had the largest positive coefficient, indicating that it was important to the model for disguising microbiomes in the diagnosis groups.

**Additional file 2**. **Figure S2:** Non-metric multidimensional scaling (NMDS) plot of Bray–Curtis dissimilarity distance index for 57 rarefied samples (18 confirmed, 17 healthy, and 22 probable pigs) on 9 swine farms. **A:** NMDS plot shows the distribution of the farms among the samples. **B:** NMDS plot shows the distribution of the age of the pigs per week among the samples. **C:** Bar plots show the distribution of the water medication from each farm. **D:** Bar plots show the distribution of the feed medication from each farm.

**Additional file 3**. **Table S1:** Total number of pigs before and after data quality filtering. Confirmed cases are pigs that showed clinical signs of *S. suis* infection and S. suis was found in the systemic sites such as blood, meninges, and/or spleen. Probable cases are pigs that showed clinical signs of *S. suis* infection but *S. suis* was not found in the any systemic site. Healthy controls are pigs without clinical signs of *S. suis* infection. *Samples that were excluded during the rarefying process. **Table S2:** Non phylogenetics alpha diversity (Observed, Shannon metrics), Reads per sample and phylogenetics (PD) Diversity matrix among the 57 rarefied samples from 18 confirmed, 17 healthy, and 22 probable cases. **Table S3:** Taxonomic contributions to Dirichlet multinomial mixture (DMM) model components identifying the important taxa that drivers the variances across the samples.** Table S4:** Top ASVs that are differently abundant between the two DMM community types as identified by ANCOM-BC.

## Data Availability

The data and materials not presented in this manuscript are available from the corresponding author upon request.

## Abbreviations

AMP: Antimicrobial peptides
ANCOM-BC: Analysis of composition of microbiomes with bias correction
ASVs: Amplicon sequence variants
DADA2: Divisive amplicon denoising algorithm 2
DMM: Dirichlet multinomial mixture
*gdh*: Glutamate dehydrogenase gene
MSA: Multiple sequence alignment
NMDS: Non-metric distance scaling
OTU: Operational taxonomic unit
PCA: Principal component analysis
PCR: Polymerase chain reaction
PERMANOVA: Permutational multivariate analysis of variance
PRDC: Porcine respiratory disease complex
PRRS: Porcine reproductive and respiratory syndrome
QIIME2: Quantitative insights into microbial ecology
*recN*: Recombination/repair protein
URT: Upper respiratory tract
