# Supplementary figures and images for: Compositional analysis of the tonsil microbiota in relationship to Streptococcus suis disease in nursery pigs in Ontario
Source: Anim Microbiome. 2022 Jan 21;4:10. doi: 10.1186/s42523-022-00162-3 (PMC8780311; doi:10.1186/s42523-022-00162-3)

A

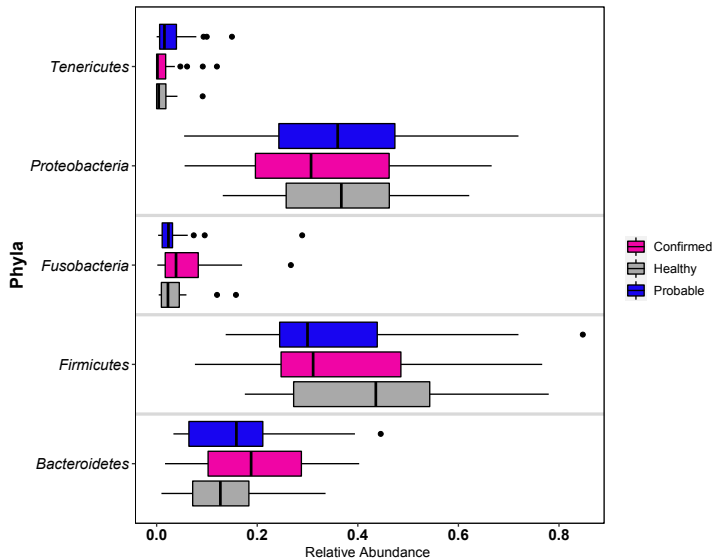

B

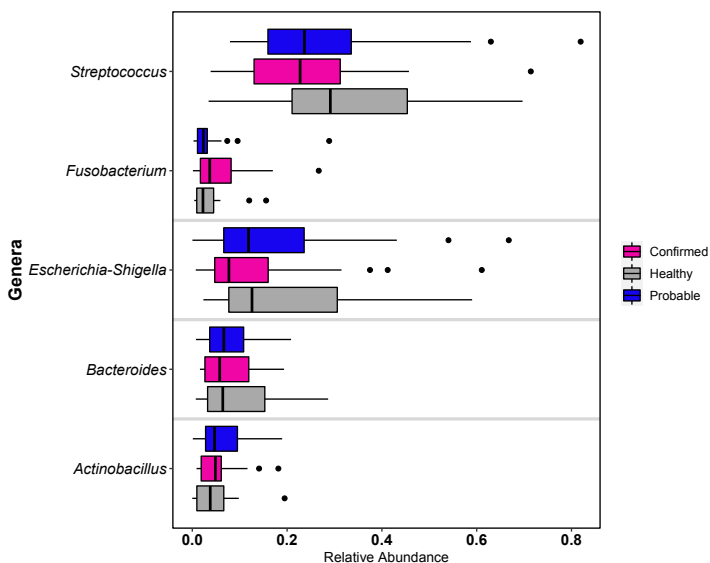

C

### Model Coefficients

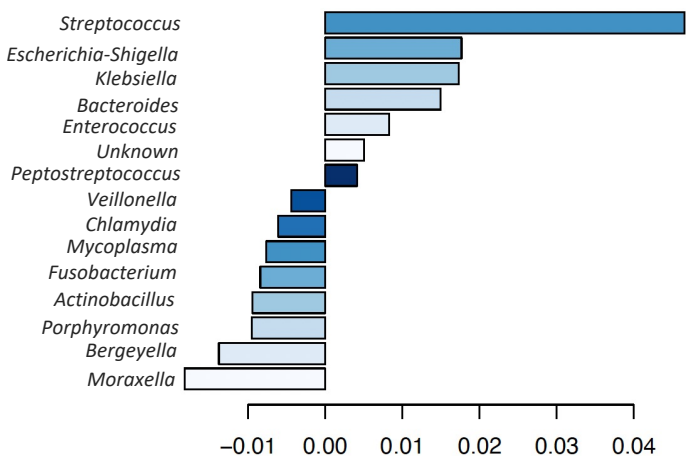

Supplement: Supplementary file 1 — Additional file 1. Figure S1: Relative abundance of tonsillar microbiota of the diagnosis groups (20 confirmed, 20 healthy, and 23 probable cases) on 9 swine farms. A: Boxplots of the top 5 phyla show that Firmicutes had the highest relative abundance across the samples. Firmicutes outliers included two confirmed cases on two different farms and one healthy pig on another farm. B: Boxplots of the relative abundance of top 5 genera show that Streptococcus had the highest relative abundance across the three diagnosis groups. Streptococcus outliers include two confirmed cases from two different farms and one healthy pig on another farm. C: Model coefficients of the PERMANOVA analysis. Only the top 15 taxa that contribute most to the tonsillar community differences are shown. The genus Streptococcus had the largest positive coefficient, indicating that it was important to the model for disguising microbiomes in the diagnosis groups. [file 42523_2022_162_MOESM1_ESM.pdf]

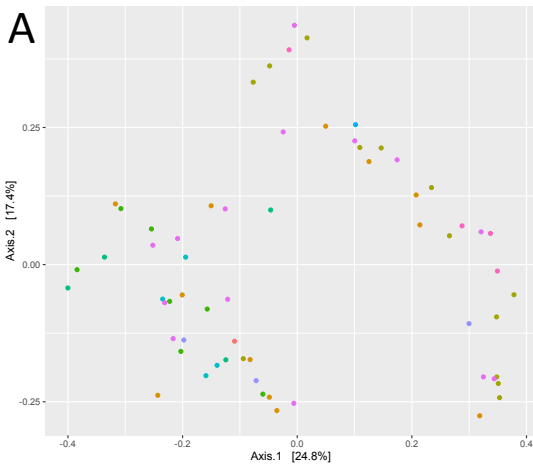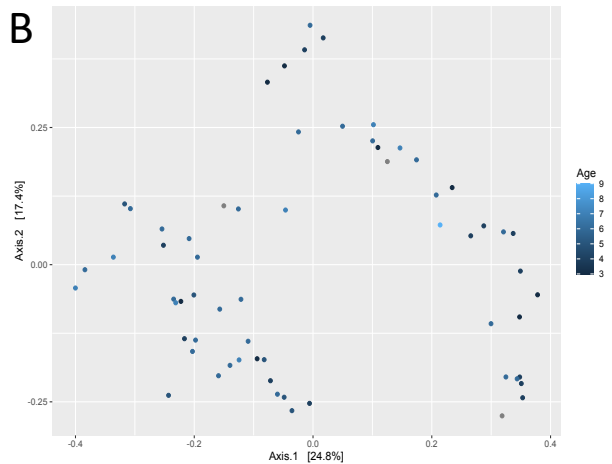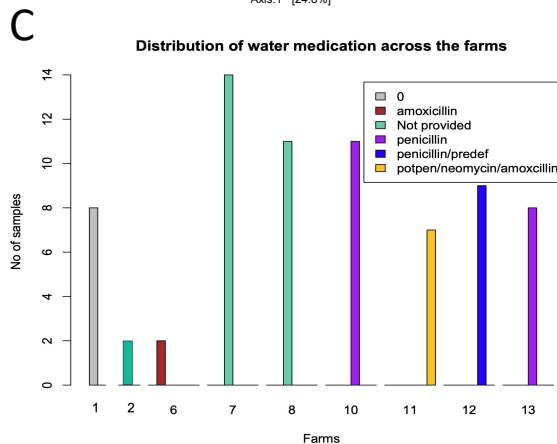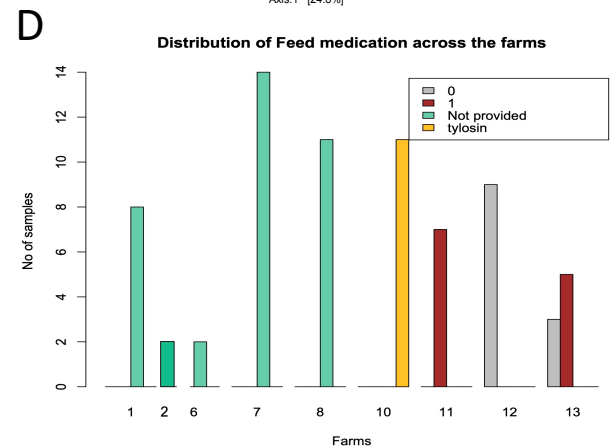

Supplement: Supplementary file 2 — Additional file 2. Figure S2: Non-metric multidimensional scaling (NMDS) plot of Bray–Curtis dissimilarity distance index for 57 rarefied samples (18 confirmed, 17 healthy, and 22 probable pigs) on 9 swine farms. A: NMDS plot shows the distribution of the farms among the samples. B: NMDS plot shows the distribution of the age of the pigs per week among the samples. C: Bar plots show the distribution of the water medication from each farm. D: Bar plots show the distribution of the feed medication from each farm. [file 42523_2022_162_MOESM2_ESM.pdf]
