# Supplementary material for: Compositional analysis of the tonsil microbiota in relationship to Streptococcus suis disease in nursery pigs in Ontario
Source: Anim Microbiome. 2022 Jan 21;4:10. doi: 10.1186/s42523-022-00162-3 (PMC8780311; doi:10.1186/s42523-022-00162-3)
Supplement: Supplementary file 3 — Additional file 3. Table S1: Total number of pigs before and after data quality filtering. Confirmed cases are pigs that showed clinical signs of S. suis infection and S. suis was found in the systemic sites such as blood, meninges, and/or spleen. Probable cases are pigs that showed clinical signs of S. suis infection but S. suis was not found in the any systemic site. Healthy controls are pigs without clinical signs of S. suis infection. *Samples that were excluded during the rarefying process. Table S2: Non phylogenetics alpha diversity (Observed, Shannon metrics), Reads per sample and phylogenetics (PD) Diversity matrix among the 57 rarefied samples from 18 confirmed, 17 healthy, and 22 probable cases. Table S3: Taxonomic contributions to Dirichlet multinomial mixture (DMM) model components identifying the important taxa that drivers the variances across the samples. Table S4: Top ASVs that are differently abundant between the two DMM community types as identified by ANCOM-BC. [file 42523_2022_162_MOESM3_ESM.docx]

**Supplemental material**

**Table S**

**Table S1:** Total number of pigs before and after data quality filtering. Confirmed cases are pigs that showed clinical signs of *S. suis* infection and *S. suis* was found in the systemic sites such as blood, meninges, and/or spleen. Probable cases are pigs that showed clinical signs of *S. suis* infection but *S. suis* was not found in any systemic site. Healthy controls are pigs without clinical signs of *S. suis* infection.

| Farm | Number of Visits | Number of pigs | | | | | |
| --- | --- | --- | --- | --- | --- | --- | --- |
|  |  | Before QC |  |  | After QC |  |  |
|  |  | **Confirmed** | **Probable** | **Healthy** | **Confirmed** | **Probable** | **Healthy** |
| 1 | **5** | **7** | **2** | **4** | **6*** | **2** | **4** |
| 2 | **1** | **0** | **1** | **0** | **0** | **1** | **0** |
| 6 | **1** | **0** | **4** | **0** | **0** | **3** | **0** |
| 7 | **2** | **7** | **4** | **4** | **7*** | **4*** | **4** |
| 8 | **1** | **1** | **1** | **2** | **1** | **1** | **2** |
| 10 | **2** | **1** | **8** | **4** | **1** | **8** | **4** |
| 11 | **1** | **3** | **2** | **2** | **3** | **2** | **2**** |
| 12 | **1** | **0** | **2** | **2** | **0** | **2** | **2*** |
| 13 | **1** | **2** | **0** | **2** | **2** | **0** | **2** |
| Total | **15** | **21** | **24** | **20** | **20** | **23** | **20** |
| Grand Total |  | **65** |  |  | **63** |  |  |

*Samples that were excluded during the rarefying process.

**Table S2** : Non phylogenetics alpha diversity (Observed, Chao1, Inverse Simpson, Shannon, Fisher and Evenness Pielou metrics), Reads per sample, and phylogenetics (PD) Diversity matrix among the 57 rarefied samples from 18 confirmed, 17 healthy, and 22 probable cases.

| Sample  ID | Diagnosis  group | Observed | Chao1 | Simpson | Shannon | Fisher | Evenness | Reads/  Sample | PD |
| --- | --- | --- | --- | --- | --- | --- | --- | --- | --- |
| F1H10 | Healthy | 2552 | 2572.04 | 1300.98 | 7.45 | 471.27 | 0.50 | 105460 | 4.31 |
| F1H11 | Healthy | 1071 | 1075.89 | 64.77 | 5.35 | 162.23 | 0.06 | 119266 | 3.59 |
| F1H12 | Healthy | 2273 | 2298.97 | 431.88 | 6.80 | 401.36 | 0.19 | 115218 | 4.21 |
| F1H8 | Healthy | 553 | 553.22 | 316.21 | 6.00 | 120.00 | 0.57 | 11917 | 2.96 |
| F7H10 | Healthy | 2902 | 2926.72 | 542.87 | 7.07 | 501.32 | 0.18 | 163224 | 5.11 |
| F7H11 | Healthy | 2685 | 2711.03 | 314.79 | 6.68 | 471.97 | 0.11 | 139024 | 4.47 |
| F7H4 | Healthy | 1769 | 1803.61 | 334.69 | 6.44 | 280.35 | 0.18 | 153910 | 4.17 |
| F7H5 | Healthy | 1035 | 1045.31 | 324.48 | 6.31 | 203.94 | 0.31 | 32416 | 5.31 |
| F8H1 | Healthy | 4140 | 4175.91 | 1311.83 | 7.64 | 752.66 | 0.31 | 183504 | 4.78 |
| F8H2 | Healthy | 2120 | 2130.67 | 512.82 | 6.78 | 368.06 | 0.24 | 116414 | 3.65 |
| F10H1 | Healthy | 2178 | 2179.20 | 401.00 | 6.80 | 391.48 | 0.18 | 101669 | 4.76 |
| F10H2 | Healthy | 1578 | 1591.92 | 242.28 | 6.27 | 273.21 | 0.15 | 87797 | 4.14 |
| F10H4 | Healthy | 2370 | 2389.60 | 398.63 | 6.76 | 426.41 | 0.16 | 110137 | 5.48 |
| F10H5 | Healthy | 3347 | 3365.12 | 1195.42 | 7.50 | 622.95 | 0.35 | 133601 | 5.88 |
| F12H1 | Healthy | 1690 | 1715.97 | 213.79 | 6.30 | 293.94 | 0.12 | 92001 | 5.65 |
| F13H1 | Healthy | 1201 | 1218.79 | 284.83 | 6.32 | 216.35 | 0.23 | 55502 | 4.46 |
| F13H2 | Healthy | 896 | 900.92 | 177.56 | 5.90 | 154.01 | 0.19 | 51622 | 4.45 |
| F10S2 | Probable | 1703 | 1703.61 | 207.31 | 6.08 | 270.17 | 0.12 | 14735 | 4.14 |
| F10S3 | Probable | 2045 | 2051.79 | 719.02 | 7.00 | 390.97 | 0.35 | 72677 | 4.80 |
| F10S4 | Probable | 1977 | 1988.06 | 706.06 | 6.97 | 359.16 | 0.35 | 87916 | 5.08 |
| F10S5 | Probable | 2590 | 2609.86 | 796.14 | 7.16 | 492.27 | 0.30 | 94380 | 5.23 |
| F10S6 | Probable | 1296 | 1310.56 | 115.29 | 5.38 | 216.78 | 0.08 | 85368 | 5.20 |
| F10S6T | Probable | 3019 | 3059.44 | 464.41 | 7.05 | 553.08 | 0.15 | 129275 | 5.72 |
| F10S7 | Probable | 2965 | 2997.07 | 877.57 | 7.34 | 583.99 | 0.29 | 93033 | 6.15 |
| F10S8 | Probable | 1665 | 1675.05 | 331.56 | 6.54 | 290.17 | 0.19 | 89786 | 4.53 |
| F11S2 | Probable | 2452 | 2499.35 | 147.19 | 6.24 | 400.71 | 0.06 | 181694 | 5.53 |
| F11S3 | Probable | 915 | 946.12 | 271.12 | 6.16 | 180.32 | 0.29 | 28644 | 5.96 |
| F12S1 | Probable | 1666 | 1686.51 | 394.30 | 6.57 | 303.10 | 0.23 | 73605 | 4.84 |
| F12S2 | Probable | 373 | 375.50 | 192.53 | 5.53 | 72.58 | 0.51 | 12305 | 3.98 |
| F1S11 | Probable | 2527 | 2550.07 | 408.68 | 6.86 | 443.46 | 0.16 | 131871 | 6.04 |
| F1S14 | Probable | 848 | 853.22 | 237.75 | 6.07 | 164.45 | 0.28 | 28373 | 4.97 |
| F2S1 | Probable | 2478 | 2489.53 | 713.19 | 7.09 | 445.21 | 0.28 | 115916 | 4.01 |
| F6S2 | Probable | 3082 | 3098.21 | 913.02 | 7.32 | 595.90 | 0.29 | 104439 | 5.67 |
| F6S3 | Probable | 2011 | 2037.46 | 364.36 | 6.74 | 371.12 | 0.18 | 83341 | 5.59 |
| F7S10 | Probable | 2921 | 2958.72 | 456.83 | 7.05 | 553.23 | 0.15 | 108066 | 6.02 |
| F7S2 | Probable | 2310 | 2329.72 | 273.99 | 6.68 | 411.51 | 0.11 | 112371 | 6.41 |
| F7S6 | Probable | 2073 | 2116.10 | 369.10 | 6.67 | 362.31 | 0.17 | 110285 | 5.12 |
| F8S2 | Probable | 3641 | 3681.52 | 821.76 | 7.44 | 736.36 | 0.22 | 102657 | 4.73 |
| F7S8 | Probable | 2471 | 2492.01 | 223.75 | 6.43 | 451.25 | 0.09 | 107333 | 5.14 |
| F7S9 | Confirmed | 2368 | 2369.50 | 989.57 | 7.29 | 475.48 | 0.41 | 68708 | 5.47 |
| F8S1 | Confirmed | 2378 | 2403.51 | 881.63 | 7.18 | 455.18 | 0.37 | 84085 | 4.59 |
| F11S1 | Confirmed | 1546 | 1581.80 | 415.04 | 6.65 | 295.51 | 0.26 | 54986 | 6.63 |
| F11S4 | Confirmed | 1052 | 1062.59 | 276.88 | 6.16 | 181.28 | 0.26 | 59879 | 4.66 |
| F11S5 | Confirmed | 1315 | 1335.41 | 499.19 | 6.63 | 248.79 | 0.37 | 48875 | 4.83 |
| F13S1 | Confirmed | 3208 | 3266.67 | 568.85 | 7.13 | 559.33 | 0.17 | 172627 | 6.53 |
| F13S2 | Confirmed | 1391 | 1403.50 | 194.43 | 6.21 | 233.72 | 0.13 | 89588 | 6.01 |
| F1S1 | Confirmed | 1176 | 1182.89 | 222.98 | 6.23 | 223.57 | 0.18 | 42810 | 3.66 |
| F1S10 | Confirmed | 3777 | 3809.52 | 1576.69 | 7.71 | 703.73 | 0.41 | 150060 | 4.73 |
| F1S12 | Confirmed | 666 | 670.03 | 210.82 | 5.88 | 124.06 | 0.31 | 26485 | 4.74 |
| F1S13 | Confirmed | 2514 | 2544.49 | 191.41 | 6.45 | 421.39 | 0.07 | 163878 | 4.90 |
| F1S6 | Confirmed | 2691 | 2691.32 | 1079.23 | 7.41 | 472.66 | 0.40 | 139841 | 4.40 |
| F1S8 | Confirmed | 2722 | 2737.62 | 1187.91 | 7.43 | 560.44 | 0.43 | 71511 | 4.34 |
| F7S3 | Confirmed | 2045 | 2082.19 | 181.67 | 6.16 | 324.17 | 0.08 | 177700 | 4.66 |
| F7S5 | Confirmed | 1801 | 1832.57 | 146.37 | 5.92 | 269.56 | 0.08 | 214655 | 3.70 |
| F7S7 | Confirmed | 2736 | 2746.80 | 696.82 | 7.28 | 517.29 | 0.25 | 101985 | 5.00 |
| F7S1 | Confirmed | 1512 | 1527.00 | 344.11 | 6.52 | 275.26 | 0.22 | 66599 | 5.31 |
| F10S1 | Confirmed | 1195 | 1195.20 | 27.72 | 6.11 | 195.14 | 0.23 | 88895 | 3.85 |

**Table S3:** Taxonomic contributions to the Dirichlet multinomial mixture (DMM) model components identifying the important taxa that drivers the variances across the samples.

| Taxa | Mean | | | Difference |
| --- | --- | --- | --- | --- |
|  |  | Community type 1 | Community type 2 |  |
| *Escherichia-Shigella* | \| 0.18901 \| \| --- \| | \| 0.08538 \| \| --- \| | \| 0.48718 \| \| --- \| | \| 0.40179 \| \| --- \| |
| *Streptococcus* | 0.10493 | 0.11727 | 0.08804 | 0.02922 |
| *Streptococcus suis* | \| 0.03083 \| \| --- \| | \| 0.03879 \| \| --- \| | \| 0.02009 \| \| --- \| | \| 0.01870 \| \| --- \| |
| *Moraxella* | \| 0.013890 \| \| --- \| | \| 0.02059 \| \| --- \| | \| 0.00667 \| \| --- \| | \| 0.01391 \| \| --- \| |
| *Bacteroides* | \| 0.03790 \| \| --- \| | \| 0.03972 \| \| --- \| | \| 0.03199 \| \| --- \| | \| 0.00773 \| \| --- \| |
| *Porphyromonas* | \| 0.01316 \| \| --- \| | \| 0.01558 \| \| --- \| | \| 0.00807 \| \| --- \| | \| 0.00751 \| \| --- \| |
| *Actinobacillus indolicus* | \| 0.00854 \| \| --- \| | \| 0.01145 \| \| --- \| | \| 0.00414 \| \| --- \| | \| 0.00730 \| \| --- \| |
| *Veillonella* | \| 0.00981 \| \| --- \| \|  \| | \| 0.01142 \| \| --- \| \|  \| | \| 0.00576 \| \| --- \| \|  \| | \| 0.00565 \| \| --- \| \|  \| |
| *Bergeyella porcorum* | 0.00680 | \| 0.00866 \| \| --- \| | \| 0.00336 \| \| --- \| | \| 0.00529 \| \| --- \| |

**Table S 4:** Top ASVs that are differently abundant between the two DMM community types as identified by ANCOM-BC.

| ASV | p-value | Scientific name |
| --- | --- | --- |
| 6ac80af4db598f1a48252bf1a0c1eef0 | 0.004101 | *Streptococcus* (24 ASVs) |
| 435da632120571a841b33dc359224c96 | 5.04E-04 | *Escherichia-Shigella* |
| a8d671f59150c8c47256d9422d0901a5 | 2.63E-02 | *Actinobacillus indolicus* |
| f0929f462ac4f42843959460823260b6 | 5.60E-03 | *Glaesserella parasuis* |
| 16b725c704ef8dd533073f55f8b55da5 | 0.0351573 | *Porphyromonas* |
| 756bebc0fff4dd239faed29986141a5d | 0 | *Trueperella pyogenes* |
| 163cae2286d5dfde556512d76df3a7c8 | 0 | *Dielma* |
| 01382fde02487202a72c3c93b6d0b500 | 0 | *Mycoplasma hyopharyngis* |
| 5027af541477bbbf3fc6685941aaa35a | 0 | *Streptococcus plurextorum* |
| 14b447516de9e770fa6aab612fc5b1b1 | 0.02794115 | *Neisseria* |
| 8c43d6b1d3ebce7eb8c8042192aabdf2 | 3.52E-02 | *Pasteurella multocida* |
